# Engineering custom morpho- and chemotypes of *Populus* for sustainable production of biofuels, bioproducts, and biomaterials

**DOI:** 10.3389/fpls.2023.1288826

**Published:** 2023-10-30

**Authors:** C. Robin Buell, Christopher Dardick, Wayne Parrott, Robert J. Schmitz, Patrick M. Shih, Chung-Jui Tsai, Breeanna Urbanowicz

**Affiliations:** ^1^ Center for Applied Genetic Technologies, Institute of Plant Breeding, Genetics, and Genomics, Department of Crop and Soil Sciences, University of Georgia, Athens, GA, United States; ^2^ Agricultural Research Service, U.S. Department of Agriculture, Kearneysville, WV, United States; ^3^ Department of Genetics, University of Georgia, Athens, GA, United States; ^4^ Department of Plant and Microbial Biology, University of California, Berkeley, CA, United States; ^5^ Department of Plant Biology, University of Georgia, Athens, GA, United States; ^6^ Warnell School of Forestry and Natural Resources, University of Georgia, Athens, GA, United States; ^7^ Center for Complex Carbohydrate Research, Department of Biochemistry and Molecular Biology, University of Georgia, Athens, GA, United States

**Keywords:** bioproducts, poplar, cell-type specificity, genome engineering, morphotype

## Abstract

Humans have been modifying plant traits for thousands of years, first through selection (i.e., domestication) then modern breeding, and in the last 30 years, through biotechnology. These modifications have resulted in increased yield, more efficient agronomic practices, and enhanced quality traits. Precision knowledge of gene regulation and function through high-resolution single-cell omics technologies, coupled with the ability to engineer plant genomes at the DNA sequence, chromatin accessibility, and gene expression levels, can enable engineering of complex and complementary traits at the biosystem level. *Populus* spp., the primary genetic model system for woody perennials, are among the fastest growing trees in temperate zones and are important for both carbon sequestration and global carbon cycling. Ample genomic and transcriptomic resources for poplar are available including emerging single-cell omics datasets. To expand use of poplar outside of valorization of woody biomass, chassis with novel morphotypes in which stem branching and tree height are modified can be fabricated thereby leading to trees with altered leaf to wood ratios. These morphotypes can then be engineered into customized chemotypes that produce high value biofuels, bioproducts, and biomaterials not only in specific organs but also in a cell-type-specific manner. For example, the recent discovery of triterpene production in poplar leaf trichomes can be exploited using cell-type specific regulatory sequences to synthesize high value terpenes such as the jet fuel precursor bisabolene specifically in the trichomes. By spatially and temporally controlling expression, not only can pools of abundant precursors be exploited but engineered molecules can be sequestered in discrete cell structures in the leaf. The structural diversity of the hemicellulose xylan is a barrier to fully utilizing lignocellulose in biomaterial production and by leveraging cell-type-specific omics data, cell wall composition can be modified in a tailored and targeted specific manner to generate poplar wood with novel chemical features that are amenable for processing or advanced manufacturing. Precision engineering poplar as a multi-purpose sustainable feedstock highlights how genome engineering can be used to re-imagine a crop species.

## 1 Introduction

Domestication and breeding efforts have shown that selection of specific plant architecture traits across a wide array of plant species, both annuals and perennials, results in improved traits for human use, either for food, feed, or fuel (Ragauskas et al., 2006; Hollender and Dardick, 2015). Similarly, selective breeding can yield distinct chemotypes of crops with desired chemical profiles or compositions (MacKay et al., 1997; Sattler et al., 2010). With advances in biotechnology and genome engineering, existing knowledge of developmental and metabolic pathways can provide the foundation for strategic and multiplexed engineering of plant architecture and metabolism on a genome and epigenome scale. For example, an elegant but simple experiment changed the architecture of solanaceous crops to be more suited to urban agriculture, converting a vine to a compact, early maturing plant through gene editing of only three key regulators of growth and development (Kwon et al., 2020). Likewise, through direct gene editing, tomatoes with elevated levels of γ-aminobutyric acid were developed (Waltz, 2022).

Many agricultural traits of interest are the direct result of tuning gene expression (Semel et al., 2006; Rodríguez-Leal et al., 2017), which can be manipulated on several levels. Controlling transcript abundance and/or *cis*-regulatory element (CRE) activity present effective and robust approaches to tune the expression of specific genes. Such genome engineering and synthetic biology approaches require characterized DNA parts that underlie the toolkit to modulate gene expression; however, there have been few plant-specific and plant-derived parts that have been systematically characterized, tested, and optimized. As a result, many of the parts that are used in “cutting-edge” plant synthetic biology efforts are derived from mammalian systems discovered over 30 years ago (Campbell et al., 1984). Thus, there is a great opportunity to identify new plant DNA parts that will enhance the dynamic range in which one can tune and manipulate gene expression in plants. Furthermore, our understanding of the *trans*-regulatory landscape has lagged, primarily due to a lack of scalable approaches that can systematically characterize the regulatory function of transcription factors in a systematic manner beyond just their DNA binding properties. Transcriptional effector domains are a broad and poorly characterized class of protein sequences that play a role in regulating transcription either through directly affecting transcription or indirectly modifying chromatin state. Effector screens are only now being implemented in eukaryotic systems (Tycko et al., 2020), and have been poorly characterized systematically in plants.

Advances in microfluidics and droplet chemistry birthed single-cell genomics, whereby molecular events such as transcript abundance or chromatin accessibility are measured for thousands of individual cells simultaneously (Vandereyken et al., 2023). These technologies result in the ability to assign cell type labels (e.g., epidermis, xylem, mesophyll, etc.) to individual cells that are part of a mixed cell-type population in a tissue or organ. Using single-cell genomic data we can discern the identity of each cell type, detect cell-type-specific expression of transcription factors and/or key metabolic enzymes, and identify cell-type-specific CREs required for spatial and temporal regulation of gene expression that can then be used to drive transgene expression in a cell-type-specific manner (Vandereyken et al., 2023).

For several crop species, we are on the cusp of true synthetic biology in which a design-build-test-learn cycle is used to engineer new traits and/or re-engineer existing traits. While model species such as rice and *Arabidopsis thaliana* (hereafter Arabidopsis) are powerful systems for synthetic biology approaches due to their simple genomes, rapid generation times, and ease of genome editing and transformation, they fail to provide a full spectrum of phenotypes and attributes needed to engineer plants with complex traits. Perennial trees represent a sustainable source of fixed carbon and are significant feedstocks of manufacturing processes including the pulp and paper industries, and, increasingly, the bioenergy industry. Precision genome engineering in perennial woody feedstock species would provide opportunities to further benefit the bioeconomy and the environment. One such perennial woody crop is poplar.

## 2 Populus: a key species for sustainable biofuel, bioproduct, and biomaterial production

Poplars (*Populus* sp. and hybrids) are among the fastest growing trees in temperate zones important for both carbon sequestration and global carbon cycling. As a species with rich genomics resources (Tuskan et al., 2006; Evans et al., 2014), poplar has emerged as the primary genetic model system for woody perennials (Jansson and Douglas, 2007; Douglas, 2017; Zhang et al., 2019). While there has been a heavy focus on poplar cell wall composition and its use as a biofuel feedstock (An et al., 2021; Schultz and Coleman, 2021), there has also been a burst of basic research over the last few decades that has substantially improved our understanding of poplar biology. As poplar has a long breeding cycle, improving this key tree crop using traditional breeding approaches is a decades-long process. The advent of genome engineering and synthetic biology, coupled with our increased knowledge base, provides the foundation for engineering improved poplar for use as sustainable sources of biofuels, biomaterials, and bioproducts. Key to success in the development of engineered poplar will be bespoke clones with architecture, photosynthetic, biochemistry, and ecological features that are ‘tuned’ to their use as a biofuel, biomaterial and/or bioproduct feedstock.

## 3 Morphotypes and chemotypes

Most crop species were domesticated within the last 10,000 years from wild relatives with key components of domestication, known as the domestication syndrome, being modified architecture, increased seed/fruit size, reduction in seed/fruit abscission, modified flowering time, and altered seed/fruit composition (Meyer et al., 2012). Modern breeding over the past century has further improved and diversified agronomic traits represented by current elite cultivars with high yield and improved quality traits. One major component of increased yield is due, in part, to alteration of plant architecture. For example, in maize, planting density has increased from 32,000 plants/ha in the 1930s to >70,000 plants/ha in the 2000s, facilitated by changes in leaf angle resulting in plants with more erect stature (for review see Mantilla-Perez and Salas Fernandez, 2017). In other cereals, the Green Revolution was propelled by breeding dwarf stature wheat and rice (Evenson and Gollin, 2003); further improvements in wheat, barley, and oat have resulted in more narrow and erect leaves that contribute to increased grain yield. The optimized architectures of these modern morphotypes improve light capture, photosynthetic capacity, stand density, harvest index, and product quality. Similar changes in the architecture of dicot crops is exemplified by the breeding in the 1940s and 1950s of upright bushy-type beans from prostate viney beans (Kelly, 2001). These changes in architecture led to a significant change in agronomic practices due to the ability to combine upright, bush types with increased yield and improved disease resistance.

In addition to improved morphotypes, evolution and selective breeding have resulted in chemical diversity within a species as exemplified by numerous culinary herbs (oregano, thyme; aroma) (Werker et al., 1985; Trindade et al., 2018), root crops (sweet potato; carotenoid content) (Champagne et al., 2010), cannabis (cannabinoid profiles) (De Meijer, 2014), eucalyptus (essential oil profiles) (Wallis et al., 2011), and poplars (bud exudates) (Greenaway and Whatley, 1990; Greenaway et al., 1991). This diversification has resulted in ‘tuned’ chemotypes in which individual accessions have a unique metabolite profile. For example, basil cultivars have distinct profiles of phenolics that contribute to the variation associated with its use as a flavoring herb or agent (Bajomo et al., 2022). In catnip, there are four possible stereoisomers of the cat attractant nepetalactone and different accessions have unique compositions of the four stereoisomers (Lichman et al., 2020).

Thus, historical selection and conventional breeding have converted wild species into novel morpho- and chemo-types that are not only compatible with modern agronomic production practices but also have altered compositional traits that are tuned to desired human traits whether it be food, feed, or fodder.

## 4 Enabling technologies for genome and epigenome engineering of complex traits with precision

Our understanding of the *cis*-regulatory code in plants is starting to emerge (Marand et al., 2021) thanks to studies that map accessible chromatin regions reflecting nucleosome-depleted regions. These regions are often enriched for transcription factor binding elements, enhancers and silencers. Core promoters provide the necessary sequences for transcription initiation, whereas enhancers or silencers are bound by transcription factors that exert activation or repression of gene expression, respectively (Schmitz et al., 2022). The use of Assay for Transposase Accessible Chromatin sequencing (ATAC-seq) (Buenrostro et al., 2013; Lu et al., 2017; Maher et al., 2018) across a range of plant species has shown that accessible chromatin regions are enriched at the transcriptional start site marking the core promoter, yet 33-66% of accessible chromatin regions containing enhancers and promoters were located >1kb away from their target gene (Oka et al., 2017; Lu et al., 2019; Ricci et al., 2019). The ease at detecting accessible chromatin makes it possible to identify transcription factor motifs providing a framework to begin construction of gene regulatory networks.

Application of single-cell technologies to plant systems has just begun to emerge (Ryu et al., 2021); to date, multiple single-cell gene expression studies have been published in poplar using protoplasts as well as isolated nuclei, including studies on vegetative shoot apex (Conde et al., 2022), differentiating xylem (Li et al., 2021; Tung et al., 2023), and stems (Chen et al., 2021; Xie et al., 2021; Du et al., 2023). These studies have already revealed novel information on cell-type-specific gene expression, developmental trajectories of cell types, and new insights into vascular development in a woody perennial compared to the herbaceous annual, Arabidopsis. With respect to CREs, the majority of accessible chromatin studies to date have been in bulk tissue thereby representing a diverse set of cell types. At the single cell level, single nuclei RNA-seq and single cell ATAC-seq from over 70,000 cells was used to produce an atlas of CREs in the maize genome (Marand et al., 2021). This enabled discovery of cell-type-specific CREs, cell-type-specific expression of transcription factors and their binding motifs, non-cell autonomous activity of transcription factors, and genome-wide association signals that were highly enriched in cell-type-specific CREs and for which breeders had been unknowingly selecting for cell-type-specific CREs to improve trait performance (Marand et al., 2021). Application of single-cell techniques to decipher cell-type gene expression and CREs in each crop species will be instrumental in development of genome engineering reagents that have high temporal and spatial specificity.

Efforts to characterize the genome-wide landscape of plant transcription factor binding and transcription factor effector activity is revealing the components needed to function as ‘payloads’ to deliver enzymatic or effector activity to specific regions of the genome. For example, the use of dCas9 and/or TAL effectors in fusion with histone modifying enzymes such as histone acetyltransferase, histone methyltransferases and histone demethylases has been shown to engineer epigenomes in animal and plant cells (Sgro and Blancafort, 2020; Gardiner et al., 2022). Recent development of synthetic transcriptional systems have enabled the efficient design of both synthetic transcription factors and promoters (Belcher et al., 2020). Such systems have been leveraged to tether putative effector domains from various Arabidopsis transcription factors to create fusion synthetic transcription factors that have enabled the characterization and quantitatively measurement of the regulatory nature of each effector domain (i.e., activation or repression). A recent study screened over 400 Arabidopsis putative effector domains that has unlocked a rich diversity of parts that can now be used for fine-tuning gene expression (Hummel et al., 2023). Many of these newly discovered DNA parts significantly expand the dynamic range for gene activation when compared to the state-of-the-art parts used for gene activation via dCas9-based genome engineering (e.g., VP16). Importantly, many of these effector domains most likely control transcription by affecting different facets and levels of regulation, i.e., through transcription or chromatin accessibility. There are an estimated 4,729 transcription factors in the 717 genome and a systematic screen of effector domains would provide a means to either fuse them to dCas9 or other transcription factors to modulate and better control expression of endogenous genes, which will form the underlying poplar-tailored tools for engineering various facets of morphotype and chemotype in poplar. In addition, methods for epigenome engineering in plants using a variety of approaches are emerging (Ji et al., 2018; Gardiner et al., 2022).

## 5 Engineering morphotypes

**Figure 1:**
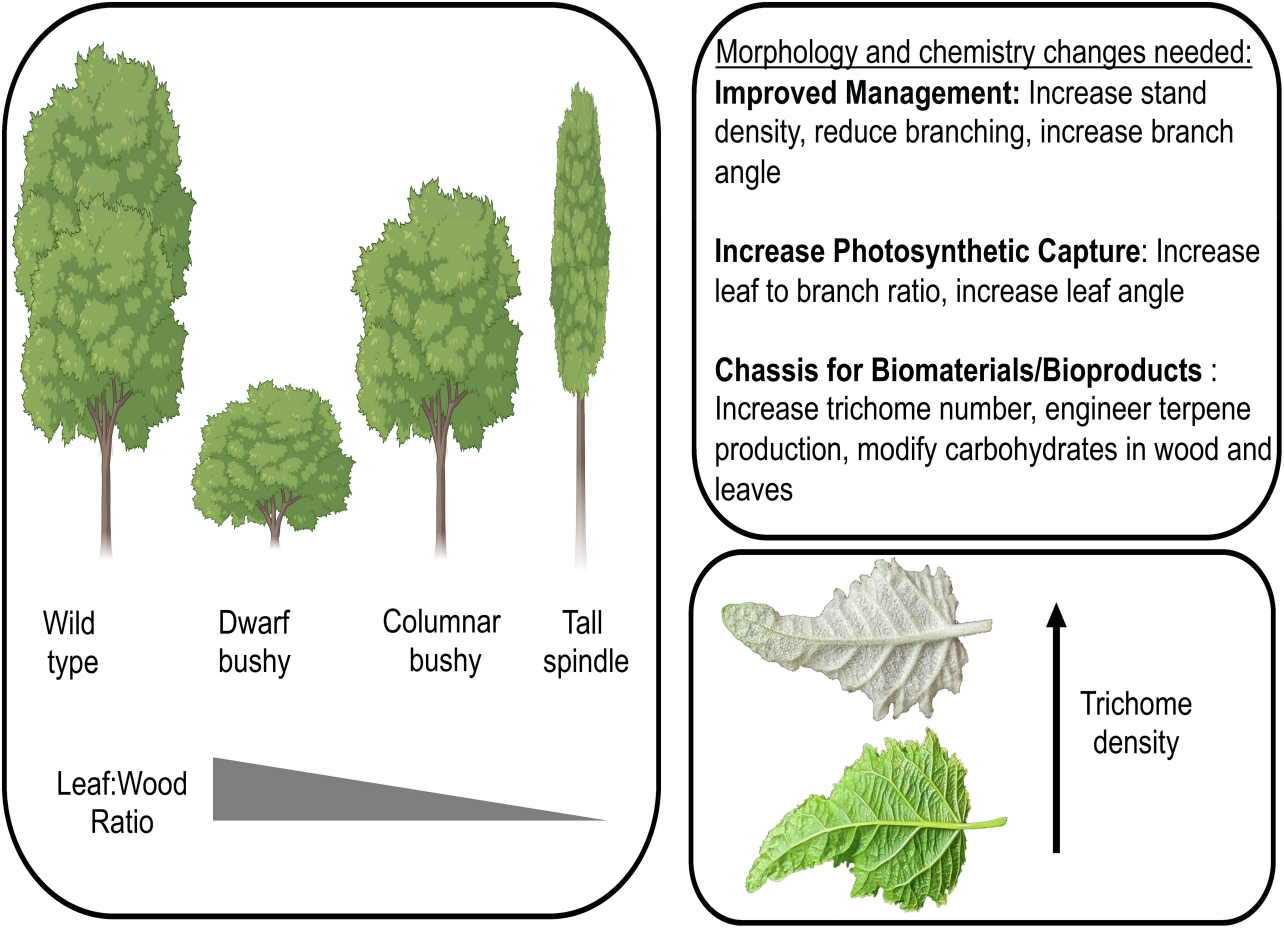
Modified poplar morphotypes can improve management practices, increase photosynthetic capture, and serve as a sustainable chassis for biofuel, biomaterial, and/or bioproduct production. Three morphotypes are depicted (dwarf bushy, columnar bushy, tall spindle) along with a leaf with increased trichome density. Morphotype images generated using Biorender.com. Leaf photograph credit: W. Patrick Bewg.

Extensive knowledge of the genetic basis of tree architecture traits provides the foundation to design novel morphotypes of poplar with modified tree architecture (**Figure 1**). Morphotypes with altered biomass potential can increase stand density, tree integrity (particularly when reducing/modifying cell wall properties), and photosynthetic capture, which can serve as foundational chassis in a biosystem design of poplar for multi-uses. The recent discovery that the non-glandular trichomes of poplar are sites of triterpene biosynthesis and accrual coupled with the discovery of transcription factors controlling trichome development (Bewg et al., 2022) provide the foundation to not only modify the density of trichomes on the leaf surface but also to hijack the trichome for synthesis of terpenoids with biofuel and bioproduct use (**Figure 1**). These chassis will have altered ratios of leaves to stem and/or trichome density in which further engineering of cell wall composition for biomaterial production and/or novel bioproducts such as precursors for drop-in fuels, thus making chemotypes that are ‘customized’ to their application and simultaneously ‘maximized’ in optimal morphotypes. As it is likely that the initial morphotypes/chemotypes will have pleiotropic effects, an iterative design process, in which developmental and metabolic pathways are optimized to create unique morpho-/chemotypes with tailored biomaterial/bioproduct composition, will be required. Through iterative design-build-test-learn cycles, poplar can be engineered into a multi-purpose feedstock, not only for bioenergy but also for biomaterial and bioproduct production.

Genome engineering technologies permit gene editing at the single-base level and in the case of outcrossing species such as poplar, with allelic resolution. The interspecific F1 hybrid *P. tremula* x *P. alba* INRA 717-1B4 (717 hereafter) (Mader et al., 2016) is highly amenable to *Agrobacterium* transformation (Leple et al., 1992) and CRISPR genome editing (Zhou et al., 2015). Key to this success is a custom variant database which facilitates SNP-aware guide RNA designs for mono-, bi- or multi-allelic editing (Tsai and Xue, 2015), and a recently generated haplotype-resolved, telomere-to-telomere assembly of the hybrid genome (Zhou et al., 2023). This has enabled efficient mutagenesis of multigene families, including genome duplicates and/or tandem repeat (Tsai et al., 2020; Bewg et al., 2022; Chen et al., 2023). Importantly, nearly 100% of edits are biallelic in 717 (Zhou et al., 2015; Tsai et al., 2020; Bewg et al., 2022). Efficient and specific editing along with demonstrated stability over multiple clonal generations (Bewg et al., 2018; Chen et al., 2023) suggests complex multi-gene engineering strategies are feasible in elite woody perennials that are propagated by vegetative means.

### 5.1 Tree architecture traits

Crown architecture, including branching characteristics, is a strong determinant of growth and productivity of woody biomass crops (Ceulemans et al., 1990). In temperate trees, crown architecture is the result of numerous developmental factors, some of which can be subject to environmental influence including phyllotaxy, bud dormancy (influenced by apical dominance and apical control), branch and leaf angles (influenced by light and gravity sensing), internode length, and branching pattern (Hollender and Dardick, 2015).

Branching pattern is primarily determined by prolepsis through outgrowth of overwintering buds (proleptic branching). Some species, including poplars, can also produce sylleptic branches through outgrowth of newly formed axillary buds (Wu and Hinckley, 2001; Oshima et al., 2013). Sylleptic branches increase leaf area for efficient photosynthetic light capture, and have long been proposed as a highly desirable trait in poplar ideotype breeding for biomass production (Ceulemans et al., 1990). However, the potential of this ‘ideotype concept’ (Dickmann et al., 2010) has not been fully realized. Synthetic biology approaches can facilitate development of designer poplar ideotypes to meet the growing demands of bioproducts and biomaterials​​. While the molecular regulation of sylleptic branching is not fully understood, studies from herbaceous models, fruit trees, and poplar have shed light on several transcription factors with evolutionarily conserved roles in plant architecture. A prime example is the TCP family of transcription factors, including TEOSINTE BRANCHED1 (TB1) in maize and BRANCHED1 (BRC1) in Arabidopsis, which suppress axillary bud outgrowth (Doebley et al., 1997; Aguilar-Martínez et al., 2007). Accordingly, mutations of *TB1/BRC1* and their orthologs in multiple monocot and dicot species, including poplar, promote shoot branching and alter plant architecture (Muhr et al., 2018). These and other transcription factors can be targeted to engineer designer poplar morphotypes, i.e., chassis, in which tree architecture is altered simultaneously resulting in improved photosynthetic light capture, harvest index, and metabolic pathways such as cell wall composition.

**Figure 2:**
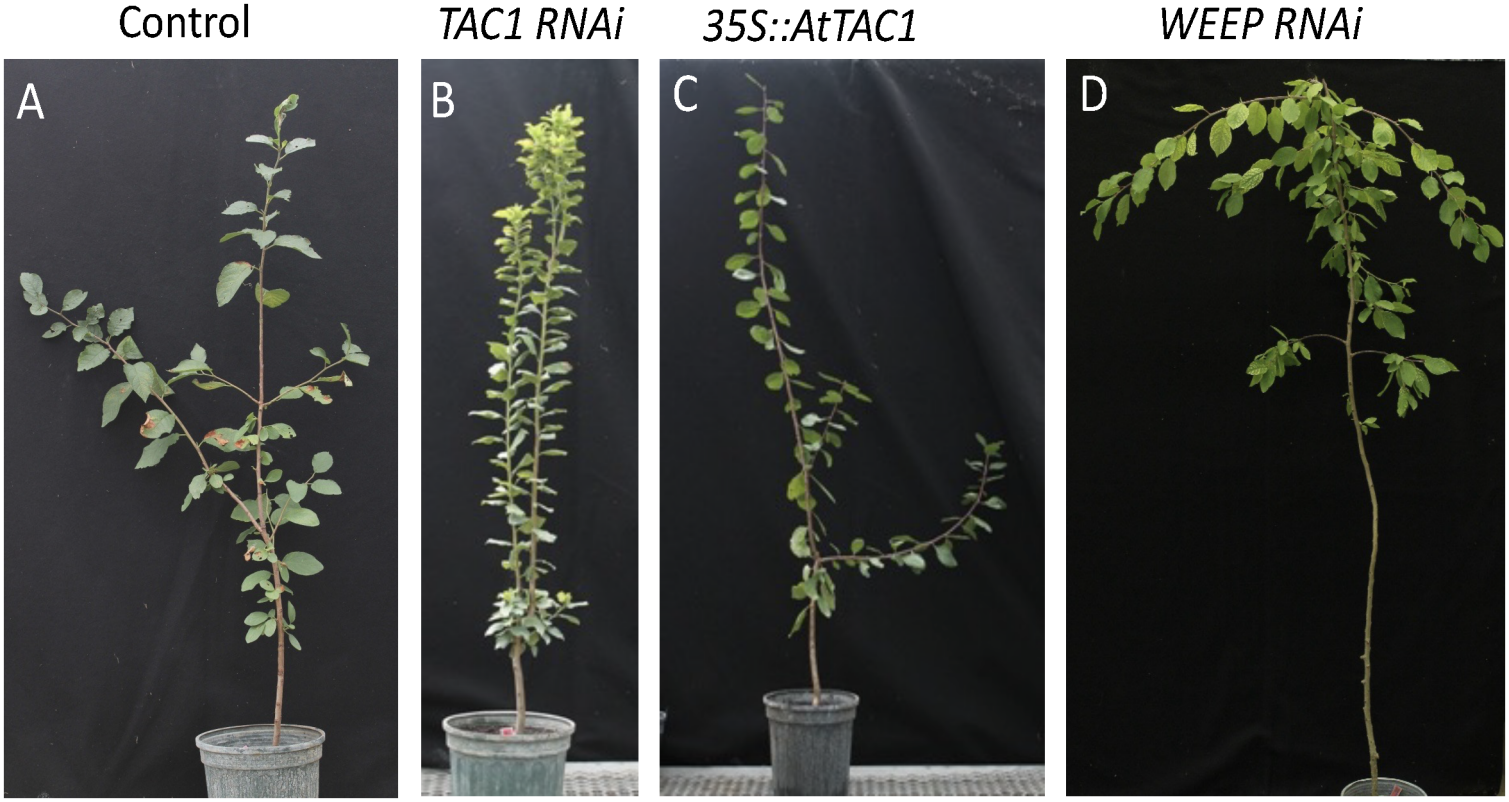
Manipulation of plum branch angle by altering known regulatory genes. **(A)** Representative control, **(B)** silencing of *TAC1* (Hollender et al., 2018b), **(C)** over-expression of Arabidopsis *TAC1*, **(D)** silencing of *WEEP* (Hollender et al., 2018a).

To design trees with altered branching patterns, it is critical to modulate branch angles to minimize competition between sylleptic branches and optimize light capture within dense canopies (Osada and Hiura, 2017). In maize, upright leaf angles improve light capture under crowded conditions (Pendleton et al., 1968). Therefore, designing a poplar morphotype needs to encompass branching patterns along with optimized branch and leaf angles. Over the past decade, significant progress has been made in understanding the genetic influences on branch growth angles. Branch angle is strongly influenced by the *IGT* family including *Tiller Angle Control 1* (*TAC1*) and *LAZY1* genes. Loss of *TAC1* in plum leads to upright branch, leaf, and flower bud angles (Dardick et al., 2013; Hollender et al., 2018b). The TAC1 pathway is known to be modulated by photosynthetic signals, suggesting a direct connection between plant architecture and light use efficiency (Waite and Dardick, 2018). In contrast, loss of *LAZY1* expression leads to horizontally oriented branches and downward oriented leaves. Transgenic poplar overexpressing *TAC1* showed increased branch angle, whereas overexpression of *LAZY1* decreased the branch angle (Xu et al., 2017). Loss of *WEEP* expression leads to downward shoot growth angles in peach and plum (**Figure 2**) and steeper root angles in Arabidopsis and its orthologue EGT2 controls root gravity responses in wheat and barley (Hollender et al., 2018a; Kirschner et al., 2021). Manipulation and fine tuning of *IGT* family genes in combination with branching genes will allow for the fine-tuning of chassis designs to optimize biomass.

Overall tree size is another critical component of optimized poplar chassis for different biomass and biomaterial applications. Smaller trees enable simpler management strategies that are more amenable to mechanization. Tree height is influenced by a combination of branching patterns and stem elongation. Elongation is controlled by cell expansion, predominantly regulated by gibberellic acid (GA) via the GA receptor Gibberellin Insensitive Dwarf 1 (GID1). GID1 is negatively regulated by DELLA proteins that mediate proteasomal degradation of GID1 via direct interactions with GA (Wang and Deng, 2014). Reduced *GID1* or over-expression of *DELLA* can lead to shorter internode lengths and reduced tree size (Mauriat and Moritz, 2009; Hollender et al., 2016). Alternatively, GA levels and tree size can be manipulated by altered expression of GA metabolic enzymes (Mauriat and Moritz, 2009; Jeon et al., 2016).

### 5.2 Trichomes as chemical factories for production of biofuels

Glandular trichomes are known for their ability to synthesize, store, and secrete large quantities of specialized metabolites, especially terpenoids, that function primarily in plant defense (Schuurink and Tissier, 2020). Many petroleum-derived commodity chemicals and fuels can be alternatively derived from terpenoids. Thus, trichomes offer a unique opportunity to hijack a natural tissue for production of targeted compounds as not only are there large pools of precursors to drive their biosynthesis, but the final molecules are naturally sequestered from other cells and organs. Non-glandular trichomes are present in a broad range of plant taxa, including Arabidopsis and *Populus*, and they synthesize and store predominantly phenolics but do not possess secretory abilities (Payne, 1978; Karabourniotis et al., 2020). We recently showed that poplar non-glandular trichomes are also sites of triterpene biosynthesis and accrual (Bewg et al., 2022).

Trichome initiation and development are under strict spatiotemporal regulation, involving the ‘MBW’ transcription activation complex of R2R3-MYB, bHLH and WD40 proteins (Maes et al., 2008; Zhao et al., 2008). Previously, activation-tagging identified *PtaMYB186* as a positive regulator of trichome development in 717 (Plett et al., 2010). PtaMYB186 is orthologous to Arabidopsis AtMYB106 involved in trichome branching (Folkers et al., 1997) and cuticle development (Oshima et al., 2013). This clade of *MYB* family is expanded in poplar, with four members derived from whole-genome and tandem duplications. Gene editing of *PtaMYB186* and its paralogs *PtaMYB138* and *PtaMYB38* resulted in glaborous plants with eight edited alleles, two more than anticipated due to unexpected duplications in one of the 717 subgenomes (Bewg et al., 2022; Zhou et al., 2023). The only exception is KO-27 which retains two wild-type (unedited) alleles with greatly reduced trichome density. The data together with previous findings of increased trichome density by *PtaMYB186* overexpression (Plett et al., 2010) support the premise that trichome density can be fine-tuned in a dose-dependent manner by manipulation of *MYB* copy number and/or expression. Interestingly, analysis of solvent extractable leaf cuticular wax revealed a complete absence of triterpenes in the glabrous mutants, while β-sitosterol was significantly reduced (Bewg et al., 2022). Reduced accrual of wax components may reflect a greatly reduced surface area due to loss of trichomes. This is the case for β-sitosterol as its levels were unchanged in whole leaf analysis. However, triterpenes remained absent in whole leaves of the mutants, implicating poplar trichomes in triterpene biosynthesis and storage (Bewg et al., 2022).

As trichomes play multiple protective roles against feeding insects, excessive transpiration, and UV radiation (Bickford, 2016), precise targeting of one to multiple alleles by CRISPR-KO or CRISPR activation (CRISPRa) can generate a spectrum of trichome phenotypes as chassis for poplar chemotype engineering.

## 6 Engineering chemotypes

With increased knowledge of biochemistry, engineered morphotype chassis can be further customized to produce biofuels, biomaterials and bioproducts tuned to their morphology. There are several target organs in poplar with potential for sustained production of biofuels, biomaterials and bioproducts: leaves and wood.

### 6.1 Trichome and leaf cuticle

Chemotype engineering of heterologous, high-value terpenes in a trichome and/or cuticle over-producing morphotype would yield a customized chemo-/morphotype. One candidate for chemotype engineering of trichomes and cuticles is the sesquiterpene, bisabolene, which has been engineered for its ability to be converted to bisabolane, a biodiesel (Peralta-Yahya et al., 2011). Rewiring plant metabolism to over accumulate bisabolene requires three strategies. First, sesquiterpenes like triterpenes are primarily produced through the mevalonate pathway, and use of feedback-insensitive enzymes will enable increased flux through the mevalonate pathway. Specifically, a truncated 3-hydroxy-3-methylglutaryl coenzyme A reductase has been widely used in such metabolic engineering efforts and shown to work in plants (Polakowski et al., 1998); by coupling this approach with the introduction of bisabolene synthase, we expect to increase production of bisabolene. Second, the plastid methyl-d-erythritol phosphate pathway can be used to increase production of bisabolene in the chloroplast (Wu et al., 2012) which has been demonstrated previously to increase production of bisabolene in *Nicotiana benthamiana* using gene stacking approaches (Shih et al., 2016). As sesquiterpenes are not natively produced via the methyl-D-erythritol phosphate pathway in the chloroplast, introducing a farnesyl diphosphate synthase into the plastid, the precursor to bisabolene, farnesyl diphosphate, can be biosynthesized and converted to bisabolene. This strategy led to an over 400% increase in production of bisabolene in *N. benthamiana*, and should be transferable to poplar as well. Third, selectively decreasing the flux to competing triterpenoid pathways by CRISPRi or gene-/allele-specific CRISPR-KO will reduce expression of key triterpene enzymes natively expressed in trichomes and/or cuticles, thereby enhancing production of the sesquiterpene of interest, bisabolene. Overall, trichomes and cuticles are an attractive platform for the sustainable production and accumulation of various terpene products of interest.

### 6.2 Cell wall modification

Fifty billion tons of carbon is assimilated by terrestrial plants annually each year (Melillo et al., 1993), representing a sustainable source of chemicals and energy. Historically, industrial applications of woody biomass have been limited mainly to bulk construction materials, viscose and lyocell textile fibers, cellulose pulps, food and feed additives, and fuel for direct combustion. The plant cell walls that make up the bulk of poplar biomass are primarily composed of cellulose, hemicellulose, and lignin (Sannigrahi et al., 2010). Cellulose provides essential mechanical support, whereas lignin and hemicellulose form additional interactions that further strengthen cell walls (Albersheim et al., 2010). Due to these hierarchical structures and interactions, plant biomass can also be used at micro-, nano-, and molecular levels for diverse applications to address current and future societal needs in the energy, environmental, materials, and manufacturing fields. Our ability to design and genetically modify the structure and interactions of the polymers in the plant cell wall is key to realizing this potential.

Progress in biorefining technologies has provided chemical and biocatalytic routes for selective valorization of leafy and woody biomass, enabling controlled fractionation to produce compounds suitable for fuel and chemical production while preserving useful glycopolymers and micro- and macro-structures (Li et al., 2022). Relative to cellulose and lignin, hemicellulose biosynthesis represents an underexplored target for altering the molecular structure and composition of cell walls. The hemicellulose xylan is a polymer composed of five-carbon (C5) sugars and is the second most abundant polysaccharide in poplar biomass after cellulose (Smith et al., 2017). It constitutes 25-35% of the cell walls of most lignocellulosic feedstocks, and roughly 10 billion metric tons of CO_2_ are assimilated by terrestrial plants into xylan each year (Smith et al., 2017). Despite their abundance, hemicelluloses have not been widely utilized to produce value-added products in modern integrated biorefineries, largely due to their complexity and structural diversity (Smith et al., 2017). Hemicelluloses are major byproducts of the multimillion tons of lignocellulose used in the production of viscose, paper products and fuels. However, C5 sugars are underutilized or considered recalcitrant (Beri et al., 2020) during the production of second-generation biofuels (Van Vleet and Jeffries, 2009), resulting in the accumulation of oligosaccharide and polysaccharide byproducts. Thus, many research efforts have aimed to reduce xylan/C5 sugar content (Yan et al., 2018), rather than optimizing its structure for more facile removal/fractionation and downstream upgrading into value-added products, such as xylan-based bioplastic building blocks or pre-biotics.

**Figure 3:**
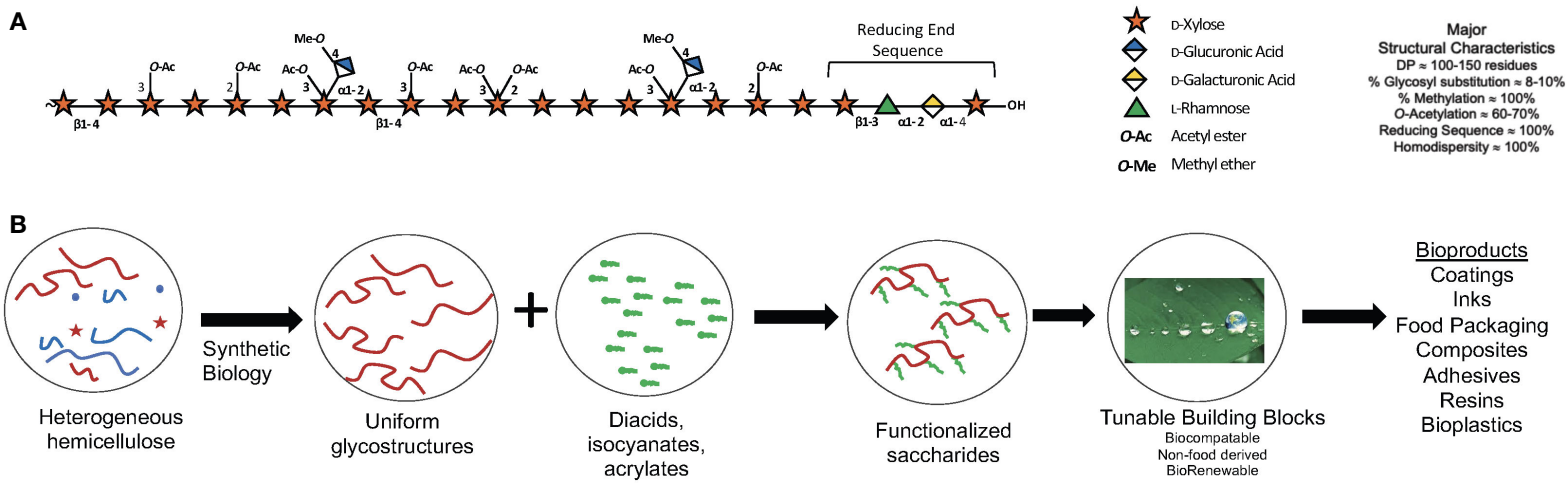
Xylan: A major component of woody cell walls. **(A)** Schematic representation *O*-acetylglucuronoxylan present in the secondary cell walls of poplar xylan highlighting abundant substituents and the characteristic reducing end sequence. The symbol nomenclature for glycans (www.ncbi.nlm.nih.gov/glycans/snfg.html) was used. **(B)** Use of synthetic biology to engineer custom cell wall chemotypes in poplar for downstream functionalization into bio-sourced materials.

Strategies can be designed to shape desirable polymer structures, increase or decrease susceptibility to chemical or enzymatic deconstruction or modification, impact wall architecture via modulation of polymer-polymer interactions, and homogenize or alter composition to reduce downstream requirements for extraction, separations, and/or purification (**Figure 3**). For poplar, integrating morphotypes that have altered physical branching patterns together with genome engineering of genes responsible for xylan synthesis and diversification in a tailored, cell-type-specific manner will enable production of material with improved chemical features amenable for processing or advanced manufacturing (Smith et al., 2017; Smith et al., 2020).

## 7 Conclusion

Through the development of high-resolution cell atlas data coupled with new genome and epigenome engineering tools, morphotypes of poplar with altered tree architecture and trichome density can be generated (**Figure 1**). These morphotypes will have altered biomass potential via increased stand density, tree integrity (particularly when modifying cell wall properties), trichome density, and photosynthetic capture, and serve as the foundational chassis. These chassis will have altered ratios of leaf to wood and/or trichome density in which we can further engineer cell wall composition for biomaterial production and/or novel bioproducts such as precursors for drop-in fuels, thus making chemotypes of poplar that are ‘customized’ to their application and simultaneously ‘maximized’ in optimal morphotypes.

## Author contributions

CB: Conceptualization, Funding acquisition, Project administration, Visualization, Writing – original draft, Writing – review & editing, Investigation, Supervision. CD: Conceptualization, Data curation, Formal Analysis, Funding acquisition, Methodology, Project administration, Visualization, Writing – original draft, Writing – review & editing. WP: Conceptualization, Funding acquisition, Writing – original draft, Writing – review & editing. RS: Conceptualization, Funding acquisition, Methodology, Writing – original draft, Writing – review & editing. PS: Conceptualization, Data curation, Funding acquisition, Methodology, Writing – original draft, Writing – review & editing. CT: Conceptualization, Data curation, Funding acquisition, Methodology, Visualization, Writing – original draft, Writing – review & editing. BU: Conceptualization, Funding acquisition, Methodology, Visualization, Writing – original draft, Writing – review & editing.
